# Correlation between ABCB1 and OLIG2 polymorphisms and the severity and prognosis of patients with cerebral infarction

**DOI:** 10.1515/med-2023-0841

**Published:** 2024-01-13

**Authors:** ChaoYing Liang, CuiYan Huang, ZhenRu Nong, SongLiang Li, MinShi Lin, ZuYe Qin

**Affiliations:** Department of Neurology, The First People’s Hospital of Qinzhou, Qinzhou City, Guangxi Zhuang Autonomous Region, 535099, China

**Keywords:** ABCB1, OLIG2, NIHSS, cerebral infarction, gene polymorphism

## Abstract

This study investigated the relationship between ATP-binding cassette sub-family B member 1 (ABCB1) and OLIG2 single nucleotide polymorphism (SNP) and neurological injury severity and outcome in cerebral infarction (CI). The neurological injury severity of 298 CI patients was evaluated by the National Institutes of Health Stroke Scale. The prognosis of CI patients at 30 days after admission was evaluated by the modified Rankin Scale. And 322 healthy people were selected as the control group. The SNPs of the ABCB1 gene (rs1045642) and OLIG2 gene (rs1059004 and rs9653711) were detected by TaqMan probe PCR, and the distribution of SNPs genotype was analyzed. SNP rs9653711 was correlated with CI. Recessive models of rs1045642 and rs9653711 were correlated with CI. The genotypes of rs1045642 and rs9653711 and genetic models were associated with CI severity. rs1045642 had no correlation with CI prognosis, while rs9653711 had less correlation. The genotype distribution and recessive model were associated with CI prognosis. SNP rs1059004 was not associated with CI severity and prognosis. Alcohol consumption, hypertension, diabetes, hyperlipidemia, and high levels of homocysteine (HCY) were independent risk factors for CI, while hypertension, hyperlipidemia, and HCY were associated with poor prognosis of CI. ABCB1 rs1045642 and OLOG2 rs9653711 are associated with CI severity.

## 1 Introduction

Cerebral infarction (CI) is a cerebrovascular disease characterized by brain necrosis and focal neuronal function defect caused by local thrombosis caused by atherosclerotic plaque and rupture of cerebral artery [1]. CI has a sudden onset, high fatality rate and disability rate, and has attracted much attention from clinicians [2]. Environmental factors of CI include diabetes mellitus, hypertension, hyperlipidemia, smoking history, etc. [3]. Currently, many studies have shown that genetic factors also affect susceptibility to CI [4–6].

ATP-binding cassette sub-family B member 1 (ABCB1) is the first ABC family transporter found in eukaryotes and encodes a P-glycoprotein (P-gp), also known as multi-drug resistance protein 1. Most studies mainly focus on drug efflux function related to tumor chemotherapy, including colorectal cancer, breast cancer, ovarian cancer, and lung cancer [7–10]. However, in earlier studies, ABCB1-encoded P-gp is a selective component of the blood–brain barrier and is arranged on the lumen plasma membrane of the brain capillary endothelium, which not only protects the central nervous system from neurotoxins but also restricts the entry of therapeutic drugs into the brain parenchyma [11,12]. In rats with ischemic stroke (IS), the concentration of ABCB1 in the ischemic side of the brain increases [13]. Numerous studies have consistently demonstrated that hypertension diabetes [14,15] and comprehensive lifestyle are established risk factors for the occurrence of IS [16,17]. Notably, a specific investigation revealed that the presence of ABCB1 gene polymorphism is closely linked to aberrant lipid metabolism, particularly in the context of hypertension [18]. Furthermore, additional research endeavors have highlighted a significant correlation between ABCB1 gene polymorphism and diastolic blood pressure (DBP) among the Chinese population or within localized regions of China, thereby suggesting a potential association with blood pressure variability [19,20]. In relation to this matter, Bochud et al. proposed a link between ABCB1 and hypertension via the renin angiotensin aldosterone system. Additionally, ABCB1 exacerbates inflammation and causes harm to ischemic tissue of different sizes by releasing endogenous inflammatory mediators. Different researchers have studied common ABCB1 gene polymorphisms. In particular, rs1045642 (C3435T) polymorphism is thought to be associated with the risk of IS, such as diabetes mellitus and dyslipidemia [21,22]. Although ABCB1 may be associated with the pathogenesis or pathological progression of IS, no case–control study has analyzed the relationship between ABCB1 gene polymorphism and the severity and prognosis of CI.

However, there is a limited number of case studies that have conducted an analysis on the pathophysiological mechanisms of recovery after CI, which involve various factors such as excitotoxicity mechanisms, inflammatory pathways, oxidative damage, ion imbalance, apoptosis, angiogenesis, neuroprotection, and neural recovery [23]. OLIG2 is a central nervous system restrictive transcription factor that plays a key role in glial progenitor cell proliferation, neural progenitor cells or their progenitor cells, and astrocytes [24]. Furthermore, an *in vivo* study has demonstrated that OLIG2 neural stem cells contribute to the recovery of spinal cord nerve injury [25]. Astrocytes are the most abundant glial cells in the central nervous system, and protect neurons in the brain under physiological conditions [26]. Most studies have shown that rs1059004 and rs9653711 polymorphisms of OLIG2 loci are associated with psychiatric diseases [27–29]. In particular, rs9653711 is associated with ischemic cerebral palsy in Chinese Han newborns [30].

In conclusion, the potential impact of ABCB1 gene polymorphism on dyslipidemia and regulation of inflammatory response, as well as the potential influence of OLIG2 gene polymorphism on the prognosis of CI through glial cell-mediated regulation of neural function, should be considered. The advancement of precision medicine has led to the widespread utilization of gene sequencing technology for disease diagnosis. However, it is worth noting that there is currently limited research investigating the association between genetic factors and susceptibility to CI, both domestically and internationally. Hence, there is an imperative need to actively investigate the etiological factors of CI and identify pertinent variables that impact patients’ neurological impairments. This research offers a foundation for personalized genetic-level diagnosis, treatment, and prevention of CI, employing a case–control study approach. This study evaluated the association of ABCB1 and OLIG2 polymorphisms with the severity and prognosis of CI.

## 2 Patients and methods

### 2.1 Patients

The present study included 298 Chinese adults with acute IS (186 males and 112 females) and 322 asymptomatic control Chinese adults (205 males and 117 females). Cases were selected from patients with CI who received neurological treatment within 24 h after onset. CI is a neurological defect caused by the obstructed circulation of cerebral blood. The diagnosis is made by a neurologist with CT, MRI, and then echocardiography and transcranial Doppler ultrasound to rule out cerebral embolism. Patients with accidental or iatrogenic stroke, transient cerebral ischemia, brain tumor, or other cerebrovascular disease were excluded. Patients should meet the following criteria: no other major disease, such as autoimmune disease, blood clotting disease, liver or kidney failure, blood disease, autoimmune or chronic family history, and no other source of embolism (aortic arch, heart, or carotid artery). The control participants were selected in a random manner from the population enrolled in a neurology health examination program. The control group was subjected to the exclusion criteria mentioned earlier, as well as the exclusion of individuals with stroke, ischemic heart disease, neurological disorders, or any severe underlying illnesses.


**Ethical statement:** All procedures performed in this study involving human participants were in accordance with the ethical standards of the institutional and/or national research committee and with the 1964 Helsinki Declaration and its later amendments or comparable ethical standards. All subjects were approved by The First People s Hospital of Qinzhou. Participants received written informed consent. If the patient was in isolation, informed consent was obtained from the next of kin or legal guardian.

### 2.2 Single nucleotide polymorphism (SNP) selection and genotyping

Based on public SNP databases including dbSNP (http://www.ncbi.nlm.nih.gov/SNP), one SNP locus of ABCB1, rs1045642 (allele frequency greater than 0.01), and two SNP loci of OLIG2, rs1059004 and rs9653711 (allele frequency greater than 0.01) were selected.

After all subjects were fasted for 12 h, peripheral blood samples were collected using EDTA anticoagulant tubes. According to the protocol provided, genomic DNA was extracted from the sample using the QIAamp DNA Blood Mini Kit (Qiagen GmbH, Germany). DNA concentrations were then measured using the Nanodrop 2000 spectrophotometer (ThermoFisher Scientific, USA). TaqMan probe fluorescence PCR was used to genotype ABCB1 and OLIG2. Different primers and probes were designed for different SNP polymorphisms of ABCB1 and OLOG2, and gene polymorphisms at different sites were detected through different channels in the reaction system. Fluorescence signals were collected using Roche LightCycler 480 II. PCR was performed under the following conditions: step 1: 95℃ for 5 min and 37℃ for 10 min. Step 2: amplification at 95℃ for 15 s, in total of 40 cycles. Step 3: 95℃ for 15 s and 72℃ for 5 min. rs1045642, sense primer: TGAATGTTCAGTGGCTCCGAG, antisense primer ACCCCTAAGGCTGACAAAGG. rs1059004 sense primer: ACGTTGGATGTCCTCACTAGAACTCATCCG, antisense primer ACGTTGGATGACGCTCTCAGGGAAAGAAGT. rs9653711 sense primer ACGTTGGATGTTTCCTTGGCTTCAGCTGCG, antisense primer ACGTTGGATGAGATTCTGCACCAAGCACTC.

### 2.3 General data and clinical biochemical analysis

General information about all subjects, including age, gender, smoking history, alcohol consumption, and body mass index (BMI), was investigated. For subjects, three consecutive measurements were taken by a professional physician using an electronic blood pressure meter. Systolic blood pressure (SBP) and DBP were defined as the average of the three measurements. Hypertension is diagnosed with SBP ≥140 mmHg and DBP >90 mmHg. Fasting blood glucose >7.0 mmol/L was recognized as diabetes mellitus. Triglyceride (TG) >2.3 mmol/L, total cholesterol (TC) >6.19 mmol/L, or high-density lipoprotein cholesterol (HDL-C) >1.0 mmol/L and low-density lipoprotein cholesterol (LDL-C) >3.4 mmol/L are considered dyslipidemia. Homocysteine (HCY) >15 μmol/L is considered to exceed the standard. The detection indexes were determined by TBA-120FR automatic biochemical analyzer (Toshiba Corporation).

### 2.4 Clinical grading and prognosis assessment of patients with CI

Neurological impairment in CI patients was assessed using the National Institutes of Health stroke scale (NIHSS). NIHSS graded CI through the examination scale of 15 neurological function items (consciousness, election observation mission, facial paralysis, limb strength, anesthesia, speech, dysarthria, and neglect), ranging from 0–15 (mild-moderate) to 16–42 (severe) [31]. The score was evaluated objectively by three professional neurologists or researchers [32]. The modified Rankin Scale (mRS) was used to evaluate the prognosis of CI patients 30 days after admission, and the patients were divided into a good prognosis group and a poor prognosis group. The poor prognosis group (mRS: 3–5 points) was defined as life dependence or death within 1 month [33].

### 2.5 Statistical analysis

Statistical analysis of general data was performed using SPSS21.0 statistical software. Measurement data were checked by normal distribution test and homogeneity test of variance, and those of normal distribution were represented by *x* ± *s*. Count data were expressed as cases (%). Measurement data were compared by Student’s test. Genotype frequency was tested by chi-square test. Binary logistic analysis assessed the correlation between the gene model and CI and prognosis, and odds ratios (OR) and 95% confidence intervals (CIs) were calculated. The risk factors and prognosis of CI were screened by unconditioned logistic regression analysis. *P* < 0.05 was considered statistically significant.

## 3 Results

### 3.1 Comparison of basic clinical data and biochemical indexes

The study included 620 subjects aged 36–94 years old. CI patients and control subjects were well matched in age and gender, with no significant differences (*P* > 0.05). The BMI of CI patients was 23.63 ± 2.53, and that of control subjects was 23.51 ± 1.84, with no statistical difference (*P* > 0.05). Drinking history (*P* ＜ 0.001), hypertension history (*P* ＜ 0.001), fasting blood glucose (*P* ＜ 0.001), blood lipid (*P* = 0.012), and HCY (*P* ＜ 0.001) were higher in CI patients than in the control group (Table 1).

**Table 1:** Clinical and biochemical characteristics of subjects

Clinical data	Control	CI	*P* value
*N*	322	298	N
Age (years)	61.2 ± 11.3	62 ± 13.5	0.35
Male	205 (63.7%)	186 (62.4%)	0.803
Smoking	70 (21.7%)	85 (28.5%)	0.063
Alcohol consumption (>30 g/day)	20 (6.2%)	37 (12.4%)	**<0.001**
BMI (kg/m^2^)	23.51 ± 1.84	23.63 ± 2.53	0.121
Hypertension	72 (22.4%)	189 (63.4%)	**<0.001**
GLU (mmol/L)	5.09 ± 1.21	6.7 ± 1.32	**<0.001**
TG (mmol/L)	1.61 ± 0.58	3.2 ± 0.82	**0.012**
TC (mmol/L)	4.8 ± 1.2	5.3 ± 1.6	0.15
HDL-C (mmol/L)	1.5 ± 0.8	1.7 ± 1.2	0.76
LDL-C (mmol/L)	2.83 ± 1.42	2.39 ± 1.11	**0.044**
HCY (μmol/L)	6.01 ± 1.80	17.32 ± 3.01	**<0.001**

Bold values indicate significant difference.

### 3.2 Frequency distribution of SNP genotypes and alleles in ABCB1 and OLIG2 genes

**Figure 1:**
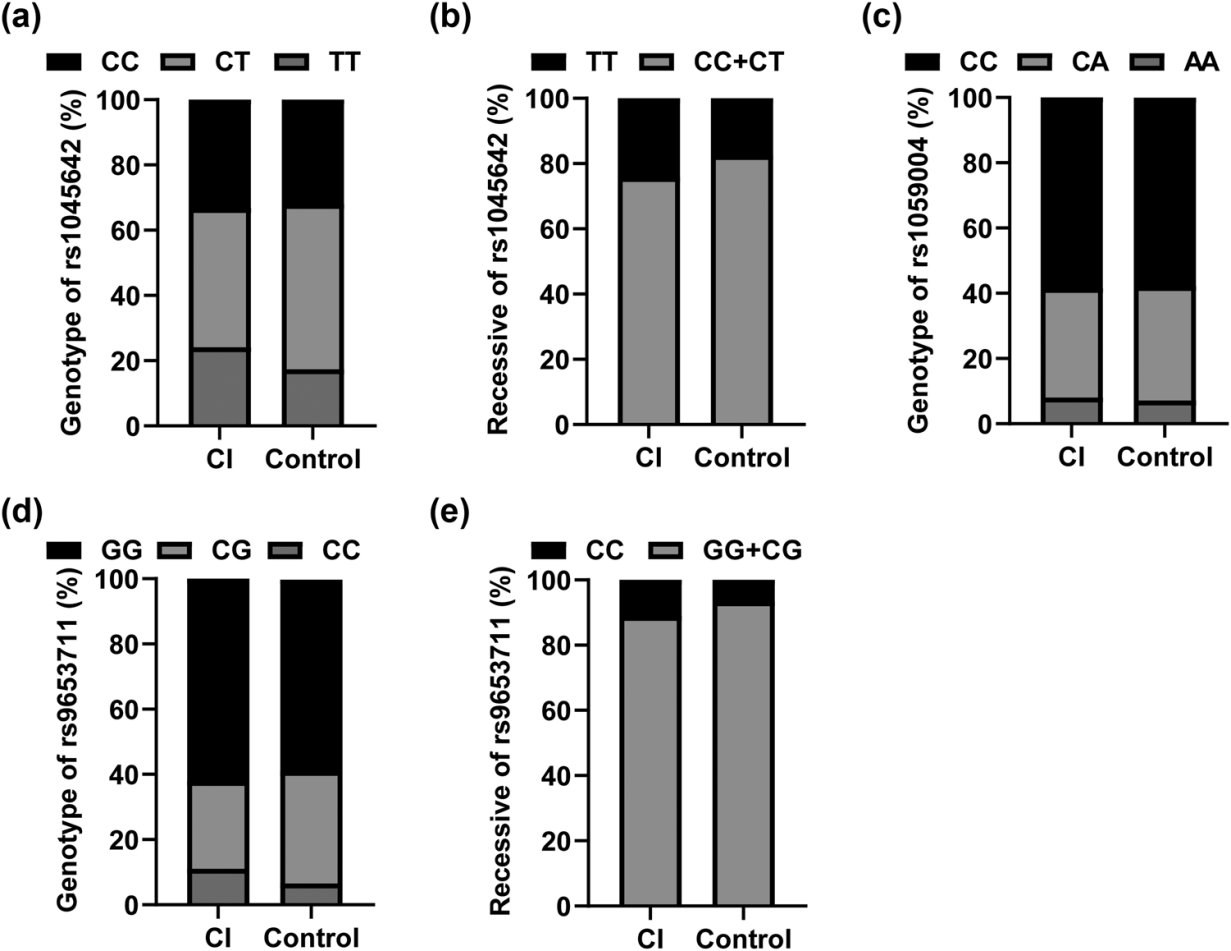
Genotype distribution of SNP locus in cerebral infarction group and control group. (a) Genotype of rs1045642; (b) Recessive of rs1045642; (c) Genotype of rs1059004; (d) Genotype of rs9653711; (e) Recessive of rs9653711.

In the control group, rs1045642, rs1059004, and rs9653711 genotype frequency distribution was consistent with Hardy–Weinberg equilibrium (*χ* ^2^ = 0.382, *P* = 0.536; *χ* ^2^ = 1.021, *P* = 0.312; *χ* ^2^ = 0.725, *P* = 0.395), indicating population representation. Table 2 shows the genotype and allele frequencies of the SNP of ABCB1 (rs1045642) and the two SNPs of OLIG2 (rs1059004 and rs9653711). As shown in Table 2, the frequency of loci rs1045642 CC, CT, and TT genotype had no statistical difference between CI patients and control group (*P* > 0.05) (Figure 1a), while the frequency of recessive model (TT/CC + CT) was higher in CI patients (*P* = 0.047, OR = 1.513, 95% CI = 1.023–2.239) (Figure 1b). Tables 3 and 4 show that the genotype, genetic model, and allele frequency of one SNP of OLIG2 (rs1059004) showed no statistical difference between CI patients and control group (*P* > 0.05) (Figure 1c). There were significant differences in the CC, CG, and GG genotypes of the SNP (rs9653711) of OLIG2 between CI patients and control group (*χ* ^2^ = 7.12, *P* = 0.028). The recessive model (CC/GG + CG) was also statistically different between the two groups (*P* = 0.047, OR = 1.785, 95% CI = 1.008–3.161) (Figure 1d and e).

**Table 2:** Genotype distribution of rs1045642 locus of ABCB1 in cerebral infarction group and control group

Genotype		CI (*n* = 298)	Control (*n* = 322)	*χ* ^2^	OR (95% CI)	*P* value
CC		99 (33.22%)	103 (31.99%)	5.628	N	0.06
CT		127 (42.62%)	163 (50.62%)			
TT		72 (24.16%)	56 (17.39%)			
Dominant	CT + TT	199 (66.78%)	219 (68.01%)	0.107	0.945 (0.676–1.323)	0.797
	CC	99 (33.22%)	103 (31.99%)			
Recessive	TT	72 (24.16%)	56 (17.39%)	4.329	1.513 (1.023–2.239)	**0.047**
	CC + CT	226 (75.84%)	266 (82.61%)			
Additive	TT	72 (24.16%)	56 (17.39%)	1.645	1.338 (0.857–2.087)	0.215
	CC	99 (33.22%)	103 (31.99%)			
Allele	T	271 (45.47%)	275 (42.70%)	0.962	1.119 (0.894–1.400)	0.331
	C	325 (54.53%)	369 (57.30%)			

*P* is the corrected value of Fisher’s test. Bold values indicate significant difference.

**Table 3:** Genotype distribution of rs1059004 locus of ABCB1 in cerebral infarction group and control group

Genotype		CI (*n* = 298)	Control (*n* = 322)	*χ* ^2^	OR (95% CI)	*P* value
CC		174 (58.39%)	186 (57.76%)	0.286	N	0.861
CA		100 (33.56%)	113 (35.09%)			
AA		24 (8.05%)	23 (7.14%)			
Dominant	CA + AA	124 (41.61%)	136 (42.24%)	0.025	0.975 (0.708–1.341)	0.935
	CC	174 (58.39%)	186 (57.76%)			
Recessive	AA	24 (8.05%)	23 (7.14%)	0.183	1.139 (0.628–2.064)	0.762
	CC + CA	274 (91.95%)	299 (92.86%)			
Additive	AA	24 (8.05%)	23 (7.14%)	0.124	1.115 (0.607–2.049)	0.758
	CC	174 (58.39%)	186 (57.76%)			
Allele	A	148 (24.83%)	159 (24.69%)	0.003	1.008 (0.778–1.304)	1
	C	448 (75.17%)	485 (75.31%)			

*P* is the corrected value of Fisher’s test.

**Table 4:** Genotype distribution of rs9653711 locus of ABCB1 in cerebral infarction group and control group

Genotype		CI (*n* = 298)	Control (*n* = 322)	*χ* ^2^	OR (95%CI)	*P* value
GG		185 (62.08%)	189 (58.70%)	7.124	N	**0.028**
CG		80 (26.85%)	112 (34.48%)			
CC		33 (11.07%)	21 (6.52%)			
Dominant	CG + CC	113 (37.92%)	133 (41.30%)	0.741	0.868 (0.629 = 1.198)	0.412
	GG	185 (62.08%)	189 (58.70%)			
Recessive	CC	33 (11.07%)	21 (6.52%)	4.033	1.785 (1.008–3.161)	**0.047**
	GG + CG	265 (88.93%)	301 (93.48%)			
Additive	CC	33 (11.07%)	21 (6.52%)	2.561	1.605 (0.896–2.877)	0.145
	GG	185 (62.08%)	189 (58.70%)			
Allele	C	146 (24.50%)	154 (23.91%)	0.057	1.032 (0.796–1.339)	0.842
	C	450 (75.50%)	490 (76.09%)			

*P* is the corrected value of Fisher’s test. Bold values indicate significant difference.

### 3.3 Relationship between SNPs loci of ABCB1 and OLIG2 genes and severity of CI

**Figure 2:**
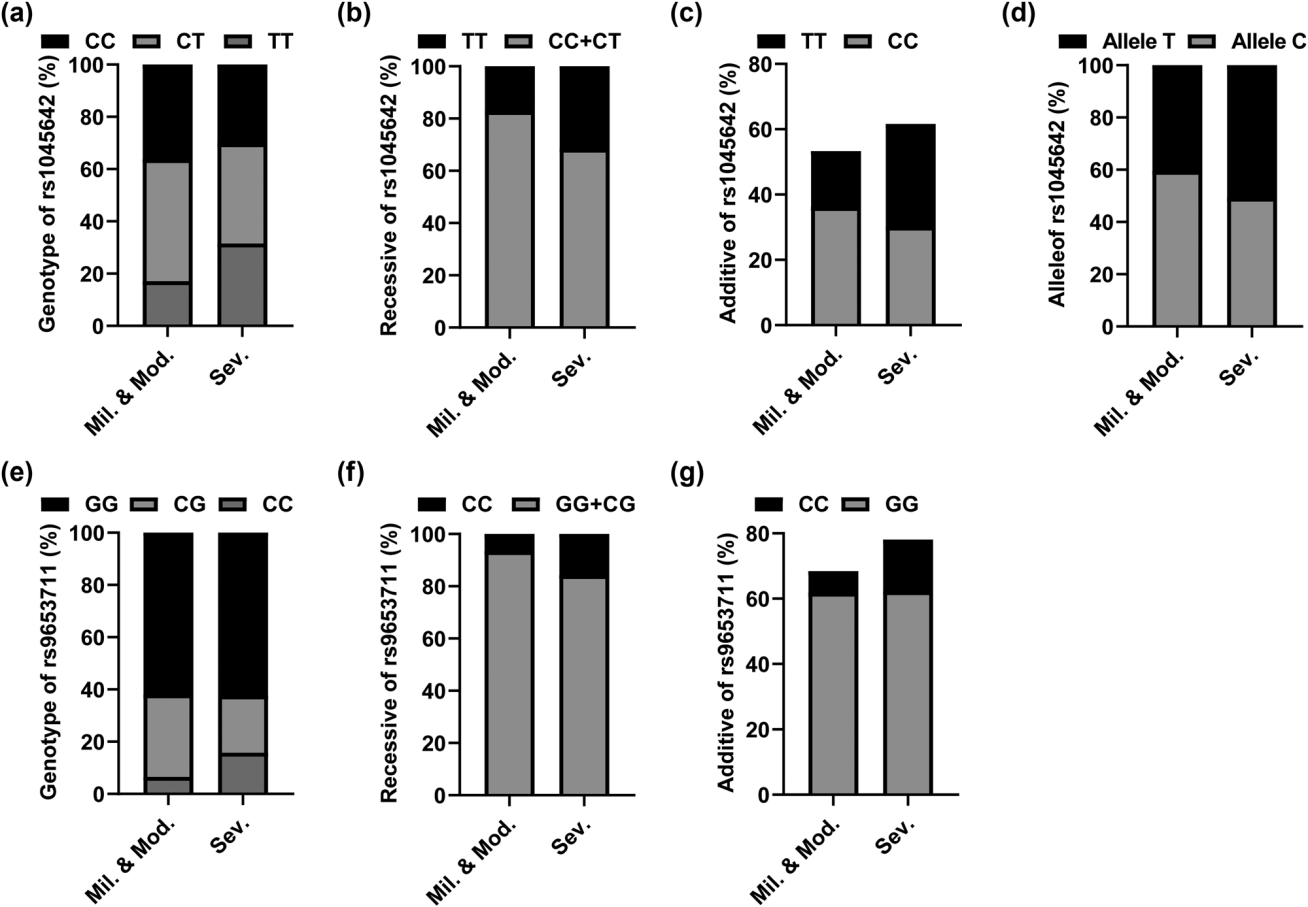
Genotype distribution of SNP locus in CI subgroups with different severities. (a) Genotype of rs1045642 in the Mid. & Mod. group and Sev. group; (b) Recessive of rs1045642 in the Mid. & Mod. group and Sev. group; (c) Additive of rs1045642 in the Mid. & Mod. group and Sev. group; (d) Allele of rs1045642 in the Mid. & Mod. group and Sev. Group; (e) Genotype of rs9653711 in the Mid. & Mod. group and Sev. group; (f) Recessive of rs9653711 in the Mid. & Mod. group and Sev. group; (g) Additive of rs9653711 in the Mid. & Mod. group and Sev. group.

To study the correlation between ABCB1 and OLIG2 and the clinical phenotype of CI, patients with CI were divided into two main subgroups according to the NIHSS. There were 152 patients with mild to moderate CI and 146 patients with severe CI. Chi-square verification and binary logistic regression analysis were used to calculate the correlation between the genotype and allele frequency of the SNP (rs1045642) of ABCB1 and the two SNPs (rs1059004 and rs9653711) of OLIG2 and the severity of CI. As shown in Table 5, the rs1045642 site of ABCB1 was closely related to the severity of CI, and the frequencies of CC, CT, and TT genotypes were statistically different between the two subgroups of CI severity (*χ* ^2^ = 8.432, *P* = 0.015) (Figure 2a). In addition, genetic models, including recessive model (TT/CC + TT), additive model (TT/CC), and allele (T/C) were statistically different between the two groups (recessive model, *P* = 0.004, OR = 2.229, 95% Cl = 1.289–3.856; additive model, *P* = 0.013, OR = 2.212, 95% CI = 1.186–4.124; allele, *P* = 0.014, OR = 1.512, 95% CI = 1.094–2.092) (Figure 2b–d). Tables 6 and 7 show that the genotype, genetic model, and allele frequency of one SNP of OLIG2 (rs1059004) had no significant correlation with the severity of CI patients (*P* > 0.05). The CC, CG, and GG genotypes of OLIG2 SNP (rs9653711) were significantly different between the two subgroups of CI severity (*χ* ^2^ = 8.252, *P* = 0.016). In addition (Figure 2e), genetic models, including recessive model (CC/GG + CG) and additive model (CC/GG), were statistically different between the two groups (recessive model, *P* = 0.016, OR = 2.655, 95% CI = 1.216–5.796; additive model, *P* = 0.037, OR = 2.376, 95% Cl = 1.071–5.269) (Figure 2f and g).

**Table 5:** Genotype distribution of rs1045642 in CI subgroups with different severities

Genotype		Mild and moderate (*n* = 152)	Severe (*n* = 146)	*χ* ^2^	OR (95%CI)	*P* value
CC		55 (36.18%)	44 (30.14%)	8.432	N	**0.015**
CT		71 (46.71%)	56 (38.36%)			
TT		26 (17.11%)	46 (31.51%)			
Dominant	CT + TT	97 (63.82%)	102 (69.86%)	1.228	1.314 (0.810–2.133)	0.272
	CC	55 (36.18%)	44 (30.14%)			
Recessive	TT	26 (17.11%)	46 (31.51%)	8.429	2.229 (1.289–3.856)	**0.004**
	CC + CT	126 (82.89%)	100 (68.49%)			
Additive	TT	26 (17.11%)	46 (31.51%)	6.322	2.212 (1.186–4.124)	**0.013**
	CC	55 (36.18%)	44 (30.14%)			
Allele	T	123 (40.46%)	148 (50.68%)	6.28	1.512 (1.094–2.092)	**0.014**
	C	181 (59.54%)	144 (49.32%)			

*P* is the corrected value of Fisher’s test. Bold values indicate significant difference.

**Table 6:** Genotype distribution of rs1059004 in CI subgroups with different severities

Genotype		Mild and moderate (*n* = 152)	Severe (*n* = 146)	*χ* ^2^	OR (95%CI)	*P* value
CC		90 (59.21%)	84 (57.53%)	0.913	N	0.667
CA		52 (34.21%)	48 (32.88%)			
AA		10 (6.58%)	14 (9.59%)			
Dominant	CA + AA	62 (40.79%)	62 (42.47%)	0.086	1.071 (0.676–1.699)	0.815
	CC	90 (59.21%)	84 (57.53%)			
Recessive	AA	10 (6.58%)	14 (9.59%)	0.911	1.506 (0.647–3.508)	0.398
	CC + CA	142 (93.42%)	132 (90.41%)			
Additive	AA	10 (6.58%)	14 (9.59%)	0.853	1.500 (0.632–3.560)	0.390
	CC	90 (59.21)	132 (90.41%)			
Allele	A	72 (23.68%)	76 (26.03%)	0.438	1.134 (0.782–1.644)	0.569
	C	232 (76.32%)	216 (73.97%)			

*P* is the corrected value of Fisher’s test.

**Table 7:** Genotype distribution of rs9653711 in CI subgroups with different severities

Genotype		Mild and moderate (*n* = 152)	Severe (*n* = 146)	*χ* ^2^	OR (95% CI)	*P* value
GG		94 (61.84%)	91 (62.33%)	8.252	N	**0.016**
CG		48 (31.58%)	32 (21.93%)			
CC		10 (6.58%)	23 (15.75%)			
Dominant	CG + CC	58 (38.16%)	55 (37.67%)	0.007	0.980 (0.613–1.564)	1
	GG	94 (61.84%)	91 (62.33%)			
Recessive	CC	10 (6.58%)	23 (15.75%)	6.365	2.655 (1.216–5.796)	**0.016**
	GG + CG	142 (93.42%)	123 (84.25%)			
Additive	CC	10 (6.58%)	23 (15.75%)	4.721	2.376 (1.071–5.269)	**0.037**
	GG	94 (61.84%)	91 (62.33%)			
Allele	C	68 (22.37%)	78 (26.71%)	1.519	1.265 (0.870–1.839)	0.253
	G	236 (77.63%)	214 (73.29%)			

*P* is the corrected value of Fisher’s test. Bold values indicate significant difference.

### 3.4 Correlation between SNPs loci of ABCB1 and OLIG2 genes and prognosis of CI

**Figure 3:**
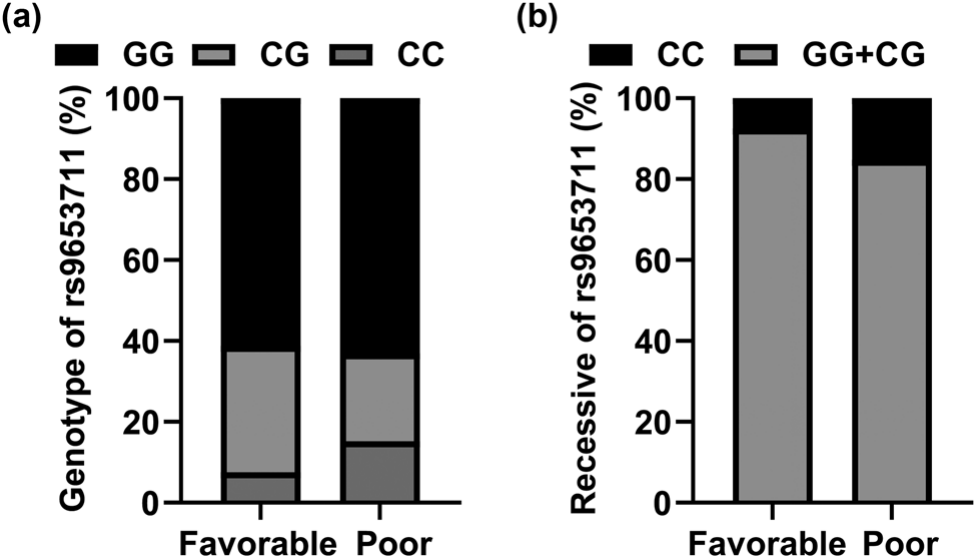
Genotypes distribution of SNP locus in CI subgroups related to prognosis. (a) Genotype of rs9653711 in the Favorable group and Poor group; (b) Recessive of rs9653711 in the Favorable group and Poor group.

To study the correlation between ABCB1 and OLIG2 and the prognosis of CI, patients with CI were divided into two subgroups: mRS Score <3 (*n* = 160) in the good prognosis group and mRS Score ≥3 (*n* = 138) in the poor prognosis group. Chi-square verification and binary logistic regression analysis were used to calculate the correlation between the genotype and allele frequencies of the SNP of ABCB1 (rs1045642) and the two SNPs of OLIG2 (rs1059004 and rs9653711) and the prognosis of CI. As shown in Table 8, the genotype, genetic model, and allele frequency of rs1045642 site of ABCB1 had no significant correlation with the prognosis of CI patients (*P* > 0.05). As shown in Tables 9 and 10, one of OLIG2’s coding SNP (rs1059004) was not significantly associated with the prognosis of CI patients (*P* > 0.05). The CC, CG, and GG genotypes of OLIG2’s other encoding SNP (rs9653711) were significantly different between the two subgroups of CI patients (*χ* ^2^ = 6.520, *P* = 0.039) (Figure 3a), and the recessive genetic model (C/GG + CG) was also significantly different between the two groups (*P* = 0.042, OR = 2.214, 95% CI = 1.046–4.684) (Figure 3b).

**Table 8:** Genotypes and allele frequencies of rs1045642 locus in CI subgroups related to prognosis

Genotype		Favorable (*n* = 160)	Poor (*n* = 138)	*χ* ^2^	OR (95% CI)	*P* value
CC		55 (34.38%)	44 (31.88%)	0.207	N	0.902
CT		67 (41.88%)	60 (43.48%)			
TT		38 (23.75%)	34 (24.64%)			
Dominant	CT + TT	105 (65.63%)	94 (68.12%)	0.207	1.119 (0.689–1.816)	0.712
	CC	55 (34.38%)	44 (31.88%)			
Recessive	TT	38 (23.75%)	34 (24.64%)	0.032	1.050 (0.617–1.786)	0.893
	CC + CT	122 (76.25%)	104 (75.36%)			
Additive	TT	38 (23.75%)	34 (24.64%)	0.13	1.118 (0.608–2.057)	0.757
	CC	55 (34.38%)	44 (31.88%)			
Allele	T	143 (44.69%)	128 (46.38%)	0.171	1.070 (0.775–1.479)	0.681
	C	177 (55.31%)	148 (53.62%)			

*P* is the corrected value of Fisher’s test.

**Table 9:** Genotypes and allele frequencies of rs101059004 locus in CI subgroups related to prognosis

Genotype		Favorable (*n* = 160)	Poor (*n* = 138)	*χ* ^2^	OR (95%CI)	*P* value
CC		90 (56.25%)	84 (60.87%)	1.089	N	0.592
CA		55 (34.38%)	45 (32.61%)			
AA		15 (9.38%)	9 (6.52%)			
Dominant	CA + AA	70 (43.75%)	54 (39.13%)	0.651	0.827 (0.520–1.313)	0.480
	CC	90 (56.25%)	84 (60.87%)			
Recessive	AA	15 (9.38%)	9 (6.52%)	1.815	0.674 (0.285–1.593)	0.401
	CC + CA	145 (90.63%)	129 (93.48%)			
Additive	AA	15 (9.38%)	9 (6.52%)	0.983	0.643 (0.267–1.547)	0.386
	CC	90 (56.25%)	84 (60.87%)			
Allele	A	85 (26.56%)	63 (22.83%)	1.108	0.818 (0.562–1.190)	0.298
	C	235 (73.44%)	213 (77.17%)			

*P* is the corrected value of Fisher’s test.

**Table 10:** Genotypes and allele frequencies of rs9653711 locus in CI subgroups related to prognosis

Genotype		Favorable (*n* = 160)	Poor (*n* = 138)	*χ* ^2^	OR (95% CI)	*P* value
GG		98 (61.25%)	87 (63.04%)	6.52	N	**0.039**
CG		50 (31.25%)	30 (21.74%)			
CC		12 (7.50%)	21 (15.22%)			
Dominant	CG + CC	62 (38.75%)	51 (36.96%)	0.101	0.927 (0.579–1.482)	0.811
	GG	98 (61.25%)	87 (63.04%)			
Recessive	CC	12 (7.50%)	21 (15.22%)	4.481	2.214 (1.046–4.684)	**0.042**
	GG + CG	148 (92.50%)	117 (84.78%)			
Additive	CC	12 (7.50%)	21 (15.22%)	3.091	1.971 (0.917–4.240)	0.091
	GG	98 (61.25%)	87 (63.04%)			
Allele	C	74 (23.13%)	72 (26.09%)	0.703	1.173 (0.807–1.705)	0.445
	G	246 (76.88%)	204 (73.91%)			

*P* is the corrected value of Fisher’s test. Bold values indicate significant difference.

## 4 Risk factors of CI morbidity and prognosis

The risk factors of CI and prognosis were analyzed by Logistic regression. As shown in Table 11, independent risk factors for CI included alcohol consumption, hypertension, diabetes, hyperlipidemia, and HCY (*P* < 0.05). Among them, the probability of CI with a drinking history, hypertension history, diabetes history, hyperlipidemia history, and high HCY level was 2.141, 6.021, 2.278, 3.956, and 2.091 times of that without the above clinical history, respectively. As shown in Table 12, the probability of poor prognosis with a history of hypertension, a history of hyperlipidemia, and a high level of HCY was 1.789, 1.685, and 1.952 times higher than that without the above clinical history, respectively.

**Table 11:** Logistic regression analysis on risk factors of cerebral infarction

Risk factors	*P*	OR (95% CI)
Smoking	0.063	1.437 (0.997–2.070)
Drinking	**0.008**	2.141 (1.212–3.780)
Hypertension	**<0.001**	6.021 (4.232–8.566)
Diabetic	**<0.001**	2.278 (1.642–3.160)
Hyperlipidemia	**<0.001**	3.956 (2.687–5.826)
HCY	**<0.001**	2.091 (1.352–3.568)

*P* is the corrected value of Fisher’s test. Bold values indicate significant difference.

**Table 12:** Logistic regression analysis on risk factors of prognosis of CI

Risk factors	*P*	OR (95% CI)
Smoking	0.063	1.681 (0.782–3.903)
Drinking	0.298	0.684 (0.547–1.098)
Hypertension	**0.05**	1.789 (1.024–3.126)
Diabetic	0.85	1.057 (0.598–1.842)
Hyperlipidemia	**0.014**	1.685 (1.132–3.283)
HCY	**0.025**	1.952 (1.045–3.425)

*P* is the corrected value of Fisher’s test. Bold values indicate significant difference.

## 5 Discussion

CI can occur at any age. With the development of society, the modern lifestyle (such as high-fat and high-glucose diet, smoking, drinking, etc.) has even induced and aggravated the incidence of CI [34]. Using SNP detection technology to study gene polymorphism and disease development from the field of molecular genetics is conducive to providing a clinical basis for prediction and treatment.

The human ABCB1 gene, located on chromosome 7, contains 29 exons and is 209 kb in length. It is an energy-dependent efflux pump that mediates bioavailability and cytotoxicity restriction [35]. So far, a variety of synonymous SNP sequences have been identified in ABCB1, among which rs1045642(C3435T) is the most widely studied [36]. ABCB1 is widely expressed in the tissue barrier, and in the blood–brain barrier, it is mainly expressed in the microvascular endothelial cells [37]. Multiple studies have confirmed that the expression of P-gp is increased in the brain of cerebral ischemia animals [13,38,39]. Studies have also found that the up-regulation of P-gp after cerebral ischemia is related to apolipoprotein E, liver X receptor, and inflammatory mediators [40–42]. P-gp is closely related to the pathophysiological mechanism of CI such as hypertension, atherosclerosis, and inflammation [18,43,44]. Therefore, it is speculated that ABCB1 gene may be a susceptibility marker for CI. At present, there are few studies on the relationship between this gene and CI. A domestic study indicated that there was no significant correlation between ABCB1 gene polymorphism and the incidence of atherosclerotic CI in Chinese Han population, while the rs1045642 site of ABCB1 had higher CC genotype LDL-C than CT genotype in female atherosclerotic CI patients [45]. A Korean study found that patients with the rs1045642 TT genotype were more likely to have a stroke 1 year after coronary intervention. In this study, rs1045642 genotype frequency of ABCB1 did not differ between CI patients and healthy subjects. However, rs1045642 gene polymorphism was associated with CI severity, the recessive model (TT/CC + CT, Fisher accurate *P* = 0.004), the additive model (TT/CC, Fisher accurate *P* = 0.013), and the allele frequency (T/C, Fisher exact *P* = 0.014). With regard to the prognosis of CI, we followed up on the prognosis of CI for 1 month and found no correlation between the gene polymorphism of this site and the prognosis (good/bad). The SNP site of the ABCB1 gene, specifically rs1045642, has been found to have the potential to decrease the expression levels of mRNA and protein products, as well as modify the affinity of the protein substrate [46]. Additionally, the 3435 C>T variant of the ABCB1 gene has been linked to elevated levels of aldosterone stimulated by angiotensin. Furthermore, studies have demonstrated the impact of the ABCB1 inhibitor cyclosporin A on the renin angiotensin aldosterone system [47,48]. In addition, alcohol consumption, hypertension, and hyperlipidemia were risk factors for CI by logistic regression analysis. Therefore, it is speculated that the T allele at rs1045642 of ABCB1 is related to these risk factors and further affects CI development, but more data are needed to support this aspect.

OLIG2 is a protein expressed in brain oligodendrocytes that plays a key role in the formation of myelin sheath, an important component of neurotransmission. The transcription factor OLIG2 is a fundamental regulator involved in the early embryonic brain’s motor neuron differentiation, as well as subsequent neural development, and the proliferation and differentiation of oligodendrocyte precursor cells in the damaged brain during individual growth and development. In a study conducted on healthy volunteers in the UK, a direct correlation was discovered between a SNP in the OLIG2 gene and a decrease in protein integrity. This finding should be included in the discussion section [49]. Therefore, OLIG2 is considered to be involved in neuronal repair after brain injury [50]. OLIG2 can significantly improve memory and cognitive impairment after transient ischemia by increasing oligodendrocyte-specific protein and brain-derived neurotrophic factor expression [51]. The human OLIG2 gene is located on chromosome 21 and contains three exons. It has been shown that in traumatic brain injury, neurons also express OLIG2 [52]. In addition, oligodendrocyte density of OLIG2^+^ EdU^+^ in the ipsilateral striate increased significantly 14 days after transient right cerebral artery occlusion in newborn animals, which may be associated with nerve repair after ischemic injury [53]. In this study, the genotype frequency of rs9653711 at OLIG2 was significantly different between CI patients and control subjects (Fisher accuracy *P* = 0.028). rs1059004 showed no difference between CI group and control group. Genotype frequency of this locus was correlated with severe CI and poor prognosis. The recessive model (CC/GG + CG, Fisher accurate *P* = 0.016) and the additive model (CC/GG, Fisher accurate *P* = 0.037) of rs9653711 were found in the mild-moderate CI and severe CI subgroups. However, no alleles (C/G) were found to be correlated with the severity of CI. Our findings provide novel insights into the association between the OLIG2 gene polymorphism and the severity of brain injury. Additionally, we have elucidated the distribution frequency of ABCB1 and OLIG2 gene polymorphisms within the population of patients with CI. Assist clinical doctors in comprehending the potential genetic mechanisms underlying CI brain injury, thereby offering a partial theoretical foundation for the prevention of CI disease progression and improvement of clinical nursing care.

## 6 Limitations

There were insufficient sample size, no correlation analysis between risk factors and SNP genotypes, and few SNP sites. In the future, sample sizes and SNPS should be expanded, or more in-depth studies should be conducted in different populations.

## 7 Conclusion

As stated in the review, the rs1045642 site of ABCB1 is correlated with the incidence of CI, and it is also found that this site is correlated with the severity of brain injury in CI, and the T allele is correlated with severe CI. rs1059004 of OLOG2 was not associated with CI, while rs9653711 was associated with the degree of brain injury and prognosis of CI. Patients carrying GG genotype were more likely to have severe CI and poor prognosis.
